# Editorial: The gut-brain axis: microbiota-driven immune modulation and its impact on neurological health

**DOI:** 10.3389/fimmu.2026.1953366

**Published:** 2026-08-21

**Authors:** Amélia M. Sarmento, Tommy Regen

**Affiliations:** 1 Faculdade de Ciências da Saúde, Universidade Fernando Pessoa, Porto, Portugal; 2 RISE-Health, Universidade Fernando Pessoa, Porto, Portugal; 3 I3S, Instituto de Investigação e Inovação em Saúde, Universidade do Porto, Porto, Portugal; 4 Institute for Molecular Medicine, University Medical Center of the Johannes Gutenberg University Mainz, Mainz, Germany; 5 Research Center for Immunotherapy (FZI), University Medical Center of the Johannes Gutenberg University Mainz, Mainz, Germany

**Keywords:** brain inflammation, gut metabolites, gut microbiota, gut-brain axes, neurological disorders

In recent years, significant attention has been given to the complex relationship between the gut and the central nervous system (CNS), better known as the gut-brain axis. This axis plays a crucial role in maintaining the homeostasis of both systems. Thereby, the two compartments are directly linked by the vagus nerve, a connection that has been already investigated in the past without, however, being entirely understood (1). More recently, the gut microbiota emerged as important modulator of the gut-brain axis. Microbial signatures and signals educate intestinal immune cells, which later egress to distant tissues, including the CNS, to influence homeostatic as well as pathogenic processes. Moreover, direct microbial products, including cellular components and metabolites, are key mediators influencing the activity of intestinal but also extra-intestinal cells (2, 3). This way, these molecules have already been implicated in CNS development, both pre- and postnatal, as well as in CNS homeostasis and disease.

This Research Topic explored recent advances in understanding the implications of the gut-brain axis, focusing on the pathogenesis of disorders such as schizophrenia, autism spectrum disorders, Alzheimer’s, and Parkinson’s. The articles included here offer new perspectives on the impact of microbiota-driven inflammation in the progression of brain diseases, while also outlining the metabolites and metabolic pathways that shape the immune mechanisms engaged. Different approaches of intestinal microbiota modulation, aiming at the restoration of brain homeostasis and the reduction of neuropsychiatric symptoms, were also covered.

## 1 Mechanisms of the gut-brain axis and neurological impairment

Five contributions provide a detailed investigation into the gut-brain axis, illustrating how microbial communities and their metabolites act as central regulators of neurological health, psychiatric stability, and pain management.

The mini-reviews from Zhou et al. and Yang et al. describe the bidirectional network linking the gut to the CNS, through neural, immune, metabolic, and endocrine pathways. Both highlight the triggering of local and systemic low-grade inflammation by dysbiosis. Locally, the disruption of the intestinal barrier (“leaky gut”) allows microbial products and inflammatory cytokines to reach the blood. These inflammatory signals reach the CNS, leading to the hyperactivation of microglia that adopt a pro-inflammatory phenotype. Then, disruption of neurotransmitter metabolism and synaptic plasticity follows, a process linked to depression, anxiety, autism, schizophrenia and neurodegenerative diseases like Alzheimer’s, Parkinson’s, and amyotrophic lateral sclerosis. Both reviews refer to different microbiota signatures associated to these neurological disorders and point out microbiota-targeted therapies (probiotics, prebiotics, and fecal microbiota transplantation) as promising strategies to restore homeostasis, reduce peripheral inflammation, and alleviate psychiatric and neurologic symptoms.

Three other contributions address specific mechanisms involved in the crosstalk between gut microbiota and the CNS.

In a rotenone-induced Parkinson’s disease mouse model (contribution by Zang et al.), the TLR4/NF-κB signalling pathway was activated in the midbrain and colon, driving significant neurological and intestinal inflammation. This process involved elevated LPS and pro-inflammatory cytokines, alongside central and peripheral α-synuclein aggregation. 16S rRNA sequencing revealed gut microbiota dysbiosis, characterized by reduced diversity and altered composition with decrease of anti-inflammatory and increase in pro-inflammatory taxa. These microbial shifts significantly correlated with motor deficits and inflammatory markers.

Yang et al. addressed tryptophan (TRP) metabolism in neurodevelopment disorders. Prenatal exposure of female juvenile rats to Poly I:C, an analogue of double stranded RNA that mimics viral infection, resulted in significant neurodevelopmental impairment of the offspring (anxiety-like behavior, impaired social interaction, and deficits in spatial learning and memory). Brain inflammation was associated to sustained microglial activation in the offspring, with up-regulation of pro-inflammatory markers and a metabolic shift of TRP metabolism to the neurotoxic kynurenine (KYN) pathway. A high KYN/TRP ratio was also found in the peripheral blood and in the intestine, associated to dysbiosis.

The review from Ouyang et al. discussed how microbial metabolites (short-chain fatty acids (SCFA), TRP derivatives and neurotransmitters) orchestrate neuroimmune crosstalk, maintaining blood-brain barrier (BBB) integrity, influencing microglial maturation and T cell differentiation. It is described how SCFA and indole derivatives promote anti-inflammatory environments through Treg expansion and aryl hydrocarbon receptor activation, and how the disruption of these pathways by dysbiosis, leads to neurologic disorders. They also discuss the importance of precision microbiota-directed interventions for restoring neuroimmune homeostasis.

## 2 Post-natal development and autism-spectrum disorders

The study by Green et al. mapped the co-evolution of the gut microbiome and T-cell ontogeny during postnatal development using a mouse model. T-cell maturation follows compartment-specific trajectories, with mucosal immune cells showing greater dynamic variation than systemic organs. The microbiome matures in sequential waves, shifting from low diversity at post-natal day 7 to an adult-like state by post-natal day 24. Integrated analysis identified modular networks linking specific taxa to functional T-cell subsets including Tregs and Th17 cells. This underscores a critical window for immune education in the post-natal period.

Prenatal exposure to valproic acid (VPA) is a risk factor for autism spectrum disorders (ASD). The original research from Liu et al. established a mechanistic link between VPA exposure and intestinal dysbiosis in a mouse model, primarily depleting SCFA-producing taxa while enriching pro-inflammatory bacterial groups. This microbial shift triggered microglial hyperactivation and increased pro-inflammatory cytokines in the brain, which, alongside oxidative stress, drove ASD-like phenotypes, including social deficits and spatial memory impairments.

The study by Wu et al. showed that immune-evasive 3KO-NSCs (CRISPR-edited human neural stem cells) significantly ameliorated ASD-like behaviours in VPA-exposed mice. The therapy suppressed central neuroinflammation by reducing hippocampal pro-inflammatory cytokines and microglial overactivation, while simultaneously restoring gut microbiota homeostasis. Specifically, treatment increased microbial diversity, enriched beneficial *Bacteroides*, and depleted pro-inflammatory *Proteobacteria*. These findings demonstrate that engineered stem cells can systemically remodel ASD pathophysiology via the gut-brain axis, constituting a potential new therapeutic strategy.

## 3 Schizophrenia and age-dependent mental disease

Three contributions provide a comprehensive analysis of the gut-brain-immune axis in patients with schizophrenia.

The original study by Ling et al. showed that elderly Chinese patients with schizophrenia exhibited significant gut mycobiota dysbiosis, characterized by increased fungal diversity and richness. These patients showed an elevated Basidiomycota/Ascomycota ratio, with enrichment of *Candida, Aspergillus, and Saccharomyces* and a marked depletion of *Purpureocillium*. A shift from *Purpureocillium*-dominant to *Candida*-dominant communities was identified in schizophrenia patients and correlated to systemic immune dysfunction involving pro-inflammatory cytokines. Species such as *P. lilacinum* and *S. cerevisiae* demonstrated exceptional diagnostic potential, positioning fungal signatures as robust non-invasive biomarkers.

Gut mycobiota dysbiosis was also present in a second study published by the same authors involving schizophrenia patients with metabolic syndrome (Ling et al.). In those patients, pathobionts like *C. albicans* and *Trichosporon asahii* were enriched, whereas beneficial *S. cerevisiae* was reduced, which correlated with systemic immune dysfunction. Furthermore, *Lodderomyces* abundance associated with elevated triglycerides, while *S. cerevisiae* correlated with reduced psychiatric symptom severity. A six-species fungal signature achieved high diagnostic accuracy for these concomitant conditions.

Approximately 30% of schizophrenia patients vs 20% healthy controls exhibit gluten sensitivity, marked by elevated anti-gliadin IgG antibodies. The original study by Dzikowski et al. highlights significant sex-related disparities: men with schizophrenia demonstrate exacerbated humoral inflammatory response, with higher concentrations of anti-gliadin and deamidated gliadin IgG compared to women. The higher humoral response to gluten was associated with higher anti-*S. cerevisiae* antibodies, presence of negative symptoms and age of onset. These findings suggest a complex interplay between sex hormones, gut barrier integrity, humoral response and psychiatric symptoms, identifying male sex as more vulnerable to immune activation in schizophrenia.

## 4 Trauma and other systemic conditions

Blunt chest trauma induces systemic inflammation and gut dysbiosis, leading to persistent cognitive deficits and anxiety-like behaviors. The original research article by Liu L. et al. demonstrated that supplementation with the probiotic *Akkermansia muciniphila* significantly ameliorates these impairments by restoring intestinal homeostasis and modulating neuroactive metabolites. Inhibition of microglial overactivation and reduction of pro-inflammatory cytokines was observed with *A. muciniphila* administration. Furthermore, it activated the BDNF/TrkB signalling pathway, enhancing synaptic plasticity and neuronal survival.

The original research from Liu L. et al. demonstrated that anxiety- and depression-like behaviours associated with chronic pancreatitis are also related to gut dysbiosis. Indeed, the study revealed that chronic pancreatitis specifically depleted beneficial *Lactobacillus* while enriched opportunistic *Helicobacter*. This microbial shift compromised intestinal and BBB integrity, elevated serum LPS and corticosterone and triggered neuroinflammation via microglial activation. Faecal microbiota transplantation confirmed that chronic pancreatitis-derived microbiota independently drives these behavioural deficits. Conversely, mixed probiotic supplementation successfully restored behavioural health and barrier function.

Liu T. et al. presented a systematic review confirming the link between vascular cognitive impairment and gut microbiota dysbiosis. They concluded that increase of *Enterobacteriaceae* and decrease of *Ruminococcus gnavus* may serve as potential diagnostic biomarkers. Literature evidence suggested that *Clostridium butyricum* and *Prevotella histicola* offer neuroprotection by reducing neuroinflammation and promoting synaptic integrity, whereas trimethylamine N-oxide exacerbates cognitive decline via NLRP3 inflammasome activation. Therapeutic interventions, including aerobic exercise, fecal microbiota transplantation and traditional Chinese medicine effectively restored microbial homeostasis, mitigating systemic inflammation and enhancing cognitive function.

The exploratory study by Ottaviano et al. identified a distinct gut microbial signature in Tuberous Sclerosis Complex (TSC) and epilepsy. Both groups exhibited significant Firmicutes depletion, primarily reducing anti-inflammatory taxa associated with butyrate production and seizure mitigation. Uniquely, the TSC group showed an enrichment of *Akkermansiaceae* and significantly fragmented microbial networks compared to healthy controls. These results suggested that intestinal dysbiosis may contribute to the neuroinflammatory processes linked to epileptogenesis in TSC.

## 5 Traditional Chinese Medicine and herbal interventions

The role of traditional Chinese medicine in the treatment and symptom mitigation of various pathologies has been progressively recognized. One possible explanation for its efficacy may be the impact on microbiota composition and function. Three studies address the role of acupuncture and herbal extracts in the treatment of neurologic disorders through microbiota modulation, providing a compelling translational bridge between traditional Chinese medicine and modern neurobiology.

As stated in the previous section, the systematic review from Liu, T. et al. points out the efficacy of acupuncture in the management of vascular cognitive impairment by reduction of symptom severity and by driving gut microbial homeostasis. Xing et al. summarized recent findings about the scientific base of acupuncture as a multi-target intervention to delay the progression of amnestic mild cognitive impairment to Alzheimer’s through modulation of the gut-brain axis. This, in turn, promotes gut barrier integrity, reduces neuroinflammation and regulates neuroendocrine pathways by balancing neurotransmitters like serotonin and GABA. Acupuncture also activates neurotrophic pathways, where microbial-derived SCFA upregulate BDNF expression, promoting synaptic plasticity and hippocampal neurogenesis.

The efficacy of herbal products has also been documented. The original research from Lian et al. in a rat model of migraine showed that administration of an extract prepared from *Angelica dahurica* Radix (Baizhi) reduced migraine-like behaviours and normalized neurochemical markers, through restoration of intestinal barrier integrity (upregulation of tight-junction proteins) and suppression of colonic inflammation. A specific enrichment of beneficial *Lactobacillus* was also observed, with increasing Firmicutes/Bacteroidetes ratio. These shifts regulated metabolic pathways like TRP and arachidonic acid metabolism, promoting neurotransmitter homeostasis. The mini-review from Liu Z. et al. focused on the role of *Schisandra chinensis* in symptom alleviation of inflammatory conditions such as ulcerative colitis, type 2 diabetes, Alzheimer’s, and depression-like behaviours. The rich content of polysaccharides and lignans in *Schisandra* rebalance microbial composition by enriching beneficial taxa while depleting pathogens, resulting in SCFA production and restoration of intestinal and BBB integrity. While animal studies highlight its therapeutic potential for metabolic and neuroinflammatory diseases, the review emphasizes that high-quality clinical trials are essential for human validation.

## 6 Conclusions

The contributions included in this Research Topic focusing on the gut-brain axis showed that dysbiosis may trigger “leaky gut” and systemic inflammation, ultimately impacting neurodevelopmental or neurodegenerative decline, like mediated by microglial hyperactivation. Evidence from Parkinson’s, ASD, and schizophrenia highlights specific mechanisms - such as TLR4/NF-κB signaling or TRP-KYN shifts - as key drivers of pathology. Interventions ranging from probiotics and engineered stem cells to acupuncture and herbal extracts show significant promise in restoring homeostasis. Ultimately, mapping these microbiota-immune-brain interactions may provide a roadmap for novel therapeutic strategies aimed to alleviate complex psychiatric and neurological symptoms, through gut microbiota modulation.
